# Ubiquitin specific peptidase 21 regulates interleukin-8 expression, stem-cell like property of human renal cell carcinoma

**DOI:** 10.18632/oncotarget.9751

**Published:** 2016-05-31

**Authors:** Liang Peng, Yi Hu, Demeng Chen, Shunchang Jiao, Shengkun Sun

**Affiliations:** ^1^ Department of Oncology, Chinese PLA General Hospital, Beijing, China; ^2^ School of Dentistry, University of California Los Angeles, Los Angeles, CA, USA; ^3^ Department of Urology, Chinese PLA General Hospital, Beijing, China

**Keywords:** USP21, renal cell carcinoma, cancer stem cell, IL-8

## Abstract

USP family proteins play essential roles in cancer cell proliferation and apoptosis and represent as candidate targets for cancer therapeutics. However, the effects and underlying mechanism of USP21 on renal cell carcinomas (RCC) remain unclear. In the present study, we investigate the effects of USP21 on proliferation, invasion and cancer stem cells (CSCs) property of RCC cell lines. As a result, siRNA-mediated depletion of USP21 inhibits cell proliferation, invasion ability and decreases the CSCs percentage of RCC cell lines. Complementarily, forced expression of USP21 leads to increase of tumorigenic properties. In addition, CSCs properties assessed by sphere formation assays demonstrated that depletion of USP21 impair the self-renewal capability of CSCs. Furthermore, decrease USP21 levels is associated with repression of interleukin 8 (IL-8), a chemokine that regulates CSCs characteristics in RCC. Mechanistically, USP21 binds to the promoter region of IL-8 and mediates transcriptional initiation. These data suggest that USP21/IL-8 could be a pair of the critical molecular targets for the development of therapeutic strategies for RCC.

## INTRODUCTION

Renal cell carcinoma (RCC) accounts for 90–95% of kidney malignancy, with estimations of 209,000 new patients and 102,000 deaths worldwide annually [1]. There are three main different morphotypes of RCC, including clear cell RCC (85–90%) papillary RCC (6–15%) and chromophobe RCC (2–5%) [2]. About 20–30% of patients already show distant metastases at the time of diagnosis [3]. Currently, surgical treatments are the only curative therapeutic approaches for early RCC [4]. However, the five-year survival rate of patients with RCC is relatively low [5]. Cancer stem-like cells (CSCs) are defined by their ability of tumor initiation, self-renewal and differentiation [6]. CSCs can survive chemotherapy and radiotherapy and are responsible for relapse after treatment and distant metastasis. Hence, it is of great importance to unmask the molecular mechanisms underlie the maintenance and regulation of CSCs in RCC.

Epigenetic regulation refers to heritable changes in gene expression that does not involve changes to the underlying DNA sequence [7–11]. USP21, which can epigenetically activate transcriptional activity of target genes, is a member of the ubiquitin specific protease (USP) family of deubiquitinating enzymes (DUBs) [12, 13]. USP21 can regulate transcriptional initiation through catalyzing the hydrolysis of the ubiquitylation of histone H2A (H2AK119ub), which represses the di- and trimethylation of H3K4 [14]. In addition, USP21 can deuibiquitinate and catalyze proteolysis of various protein substrates. For example, USP21 deubiquitinates receptor-interacting protein kinase 1 (RIP1) and repress the signaling transduction activity downstream of TNFα receptor 1 (TNFR1) and NFκB pathway [15]. Also, USP21 regulates the stability of IL-33 protein through deubiquitination and affects the transcription of p65 [16]. USP21 gene displays amplification of copy number in metastatic urothelial carcinoma [17]. However, the role of USP21 in regulating RCC and the mechanism by which USP21 affects the maintenance of CSCs has not been clarified.

We hypothesize that the expression of USP21 may be associated with tumor progression of RCC. To test this hypothesis, we firstly confirmed the upregulation of USP21 in various RCC cell lines. We then induced depletion of USP21 using siRNA in RCC cell lines (including 786-O and A-704), which significantly represses the growth, invasion ability and CSCs population of RCC cell lines. Complementarily, overexpression of USP21 promoted the tumorigenic ability of RCC cell lines. In addition, depletion of USP21 in CSCs of RCC cell lines impairs the sphere-forming ability, which is mediated through interleukin 8 (IL-8). Mechanistically, USP21 can bind to the promoter of IL-8 gene and modify the histone status for transcriptional initiation. Thus, our current study demonstrates that USP21 contributes to maintenance of CSCs in RCC.

## RESULTS

### USP21 expression is dysregulated in RCC patients

**Figure 1:**
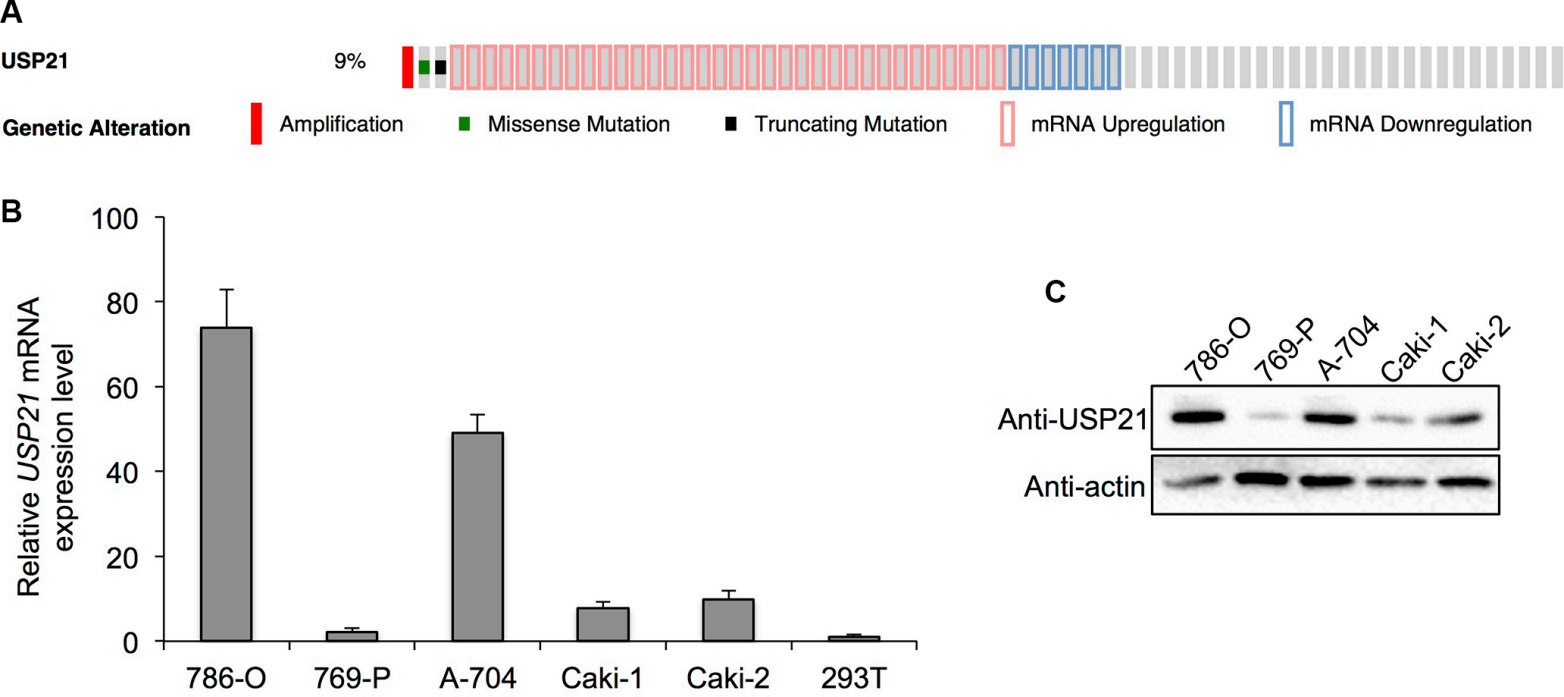
(**A**) TCGA database analysis shows that USP21 expression is dysregulated in 44 (9%) of 499 cases (**B**) qRT-PCR was used to detect the mRNA expression of USP21 in five different RCC cell lines and HEK293T cell line as normal tissue control. (**C**) Western blotting was used to detect the protein expression of USP21 in five different RCC cell lines.

To examine the expression of USP21 in RCC, we first investigated the TCGA database and found that USP21 expression is dysregulated in 44 (9%) of 499 cases (Figure 1A). We then examined the mRNA expression of *USP21* across five different RCC cell lines and normal human kidney epithelial cell line HEK293T (Figure 1B and 1C). We found that, compared with HEK293T cells, *USP21* mRNA was expressed at higher levels in all RCC cell lines, including three adenocarcinoma lines 786-O, 789-P and A-704, two clear cell carcinoma Caki-1 and Caki-2 (Figure 1B). Western blot results showed that USP21 displayed the highest expression level in 786-O and A-704 cell lines compared with other RCC cell lines (Figure 1C). Hence, these two lines were chosen for the functional characterization of USP21.

### Knockdown of USP21 decreased the cell growth, invasion and cancer stem cell percentage of 786-O cells

**Figure 2:**
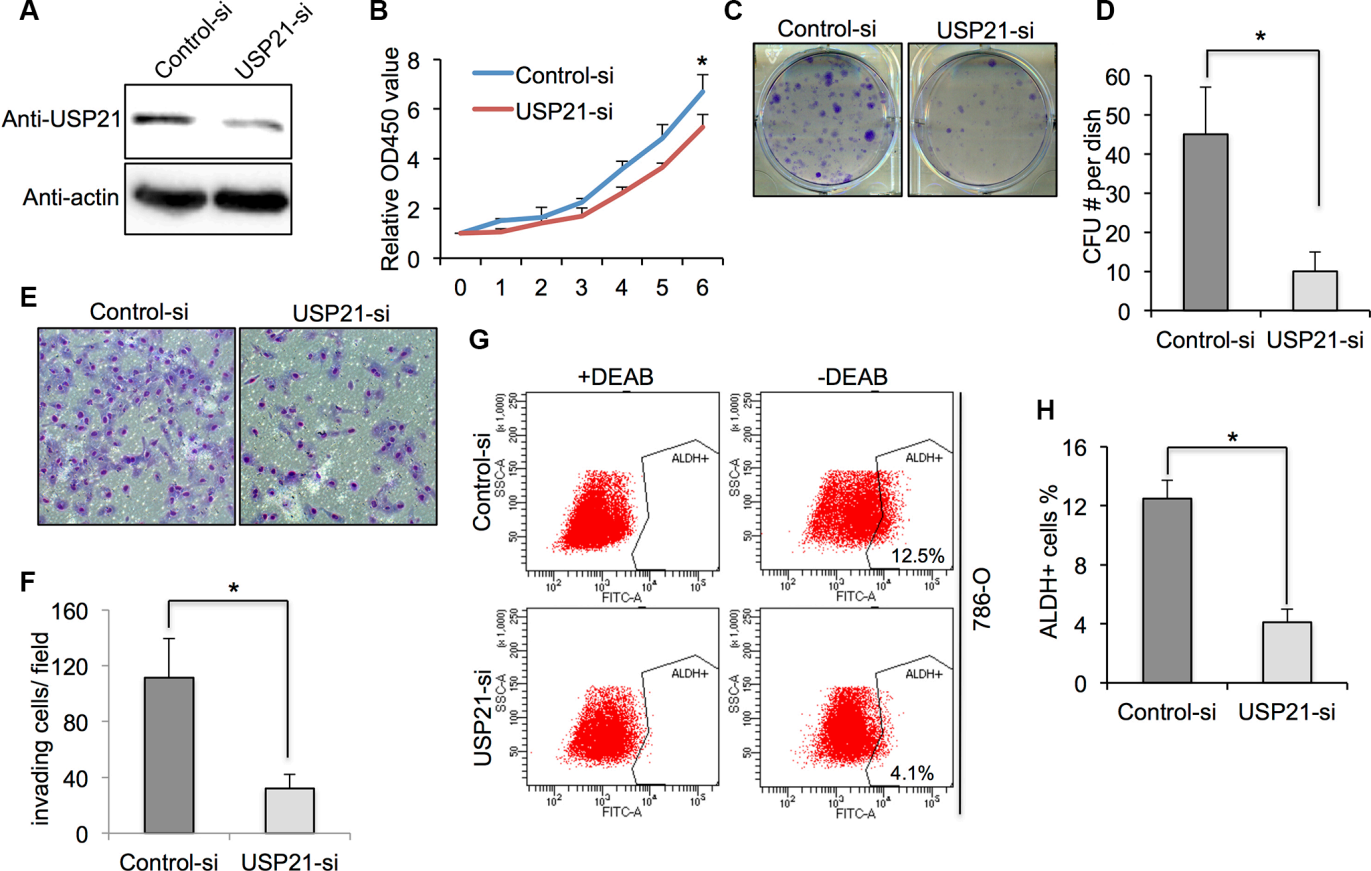
(**A**) 786-O cells transfected with control or USP21 siRNA for 48 hours were tested for the presence of USP21 protein by Western blot. Actin is shown as a loading control. (**B**) Cell proliferation of 786-O cells transfected with control or USP21 siRNA at various time points were measured by MTT assays. Data represent the mean (± s.d.) of three independent experiments, each performed in triplicate. (**C, D**) Colony formation assay in 786-O cells transfected with control or USP21 siRNA. Values are expressed as the mean ± SD. (**E, F**) Invasion assay of 786-O cells transfected with control or USP21 siRNA. (x100 in six different fields per filter). (**G, H**) ALDHhigh cell percentage in 786-O cells transfected with control or USP21 siRNA. Diethylaminobenzaldehyde (DEAB) was used to inhibit ALDH activity, to show the specificity of detection. Data represent the mean (± s.d.) of three independent experiments, each performed in triplicate.

USP21 protein expression was detected by Western blot in 786-O cells treated with either control siRNA or USP21 siRNA. We found that the expressions of USP21 protein was significantly decreased in USP21 siRNA treated cells (Figure 2A). To study the impact of USP21 on 786-O cell proliferation, we performed MTT and colony formation assays. While cell proliferation rates were comparable at early time points examined, we found that USP21 depletion led to dramatically decrease of cell proliferation 6 days after transfection (Figure 2B). Moreover, knockdown of USP21 in 786-O cells displayed significant less colonies compared to control cells (Figure 2C and 2D). To explore the functional role of USP21 on invasion in 786-O cells, we performed matrigel invasion chamber assays using cells transfected with control or USP21 siRNA. Our data revealed that knockdown of USP21 markedly reduced invasiveness of 786-O (Figure 2E and 2F). Next, to examine whether USP21 plays a role in the CSCs population in 786-O cells, we used flow cytometry approach to detect the ALDH^high^cells, which are reported as CSCs in RCC [18]. In the control cell lines, we observed 12.5% ALDH^high^ cells in the total population. In contrast, only 4.1% ALDH^high^ cells were detected in the 786-O USP21 siRNA knockdown cells (Figure 2G and 2H), suggesting the loss of a specific subpopulation of CSCs. To rule out the off-target effect of this particular siRNA, we introduced another siRNA against USP21 and found similar effects of USP21 on the tumorigenic properties of 786-O cell line (Figure S1A–S1C).

### Knockdown of USP21 decreased the cell growth, invasion and cancer stem cell percentage of A-704 cells

**Figure 3:**
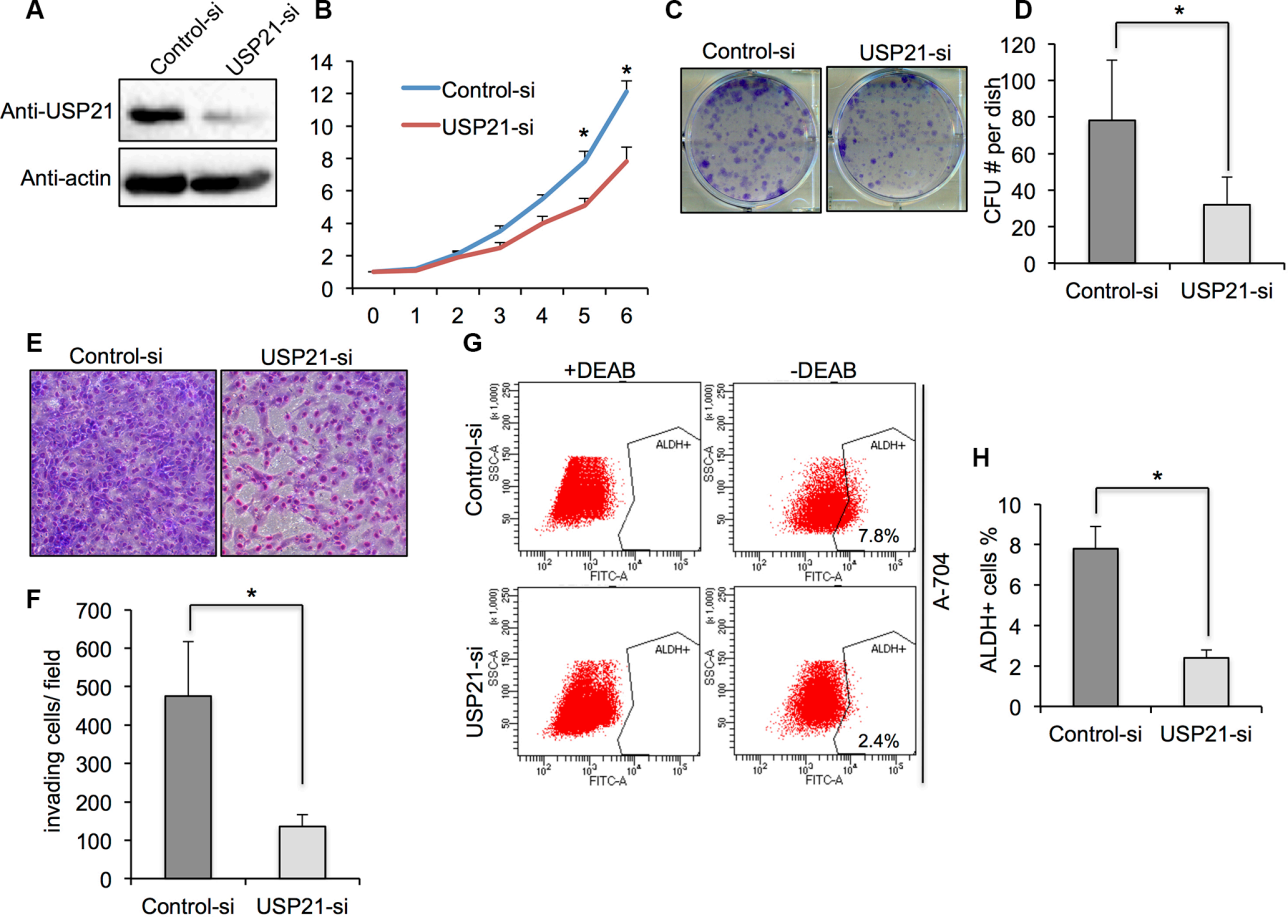
(**A**) A-704 cells transfected with control or USP21 siRNA for 48 hours were tested for the presence of USP21 protein by Western blot. Actin is shown as a loading control. (**B**) Cell proliferation of A-704 cells transfected with control or USP21 siRNA at various time points were measured by MTT assays. Data represent the mean (± s.d.) of three independent experiments, each performed in triplicate. (**C, D**) Colony formation assay in A-704 cells transfected with control or USP21 siRNA. Values are expressed as the mean ± SD. (**E, F**) Invasion assay of A-704 cells transfected with control or USP21 siRNA. (x100 in six different fields per filter). (**G, H**) ALDHhigh cell percentage in A-704 cells transfected with control or USP21 siRNA. Diethylaminobenzaldehyde (DEAB) was used to inhibit ALDH activity, to show the specificity of detection. Data represent the mean (± s.d.) of three independent experiments, each performed in triplicate.

To further confirm the effects of USP21 in renal cancer cells, we used A-704 cell line for its functional study. Western blot showed a noticeable decrease in protein expression of USP21 in A-704 cells treated with USP21 siRNA compared with control cells (Figure 3A). MTT and colony formation assays were used to demonstrate that knockdown of USP21 markedly reduced the growth at day 5 and 6 (Figure 3B) and clone-formation (Figure 3C and 3D) rate of A-704 cell lines as compared with that of control transfected cells. We observed a decrease of invasion ability in USP21 siRNA treated cell (135 cells/field) compared with control cells (475 cells/ field) (Figure 3E and 3F). We compared the number of ALDH^high^ cells in the control and USP21 siRNA A-704 cells. Similarly, our data displayed a prominently decrease in CSCs population in USP21 knockdown cells (Figure 3G and 3H). We used the second siRNA against USP21 and confirmed its role in the tumorigenic properties of A-704 cell line (Figure S1A–S1C).

### USP21 was required for CSCs self-renewal ability in RCC

**Figure 4:**
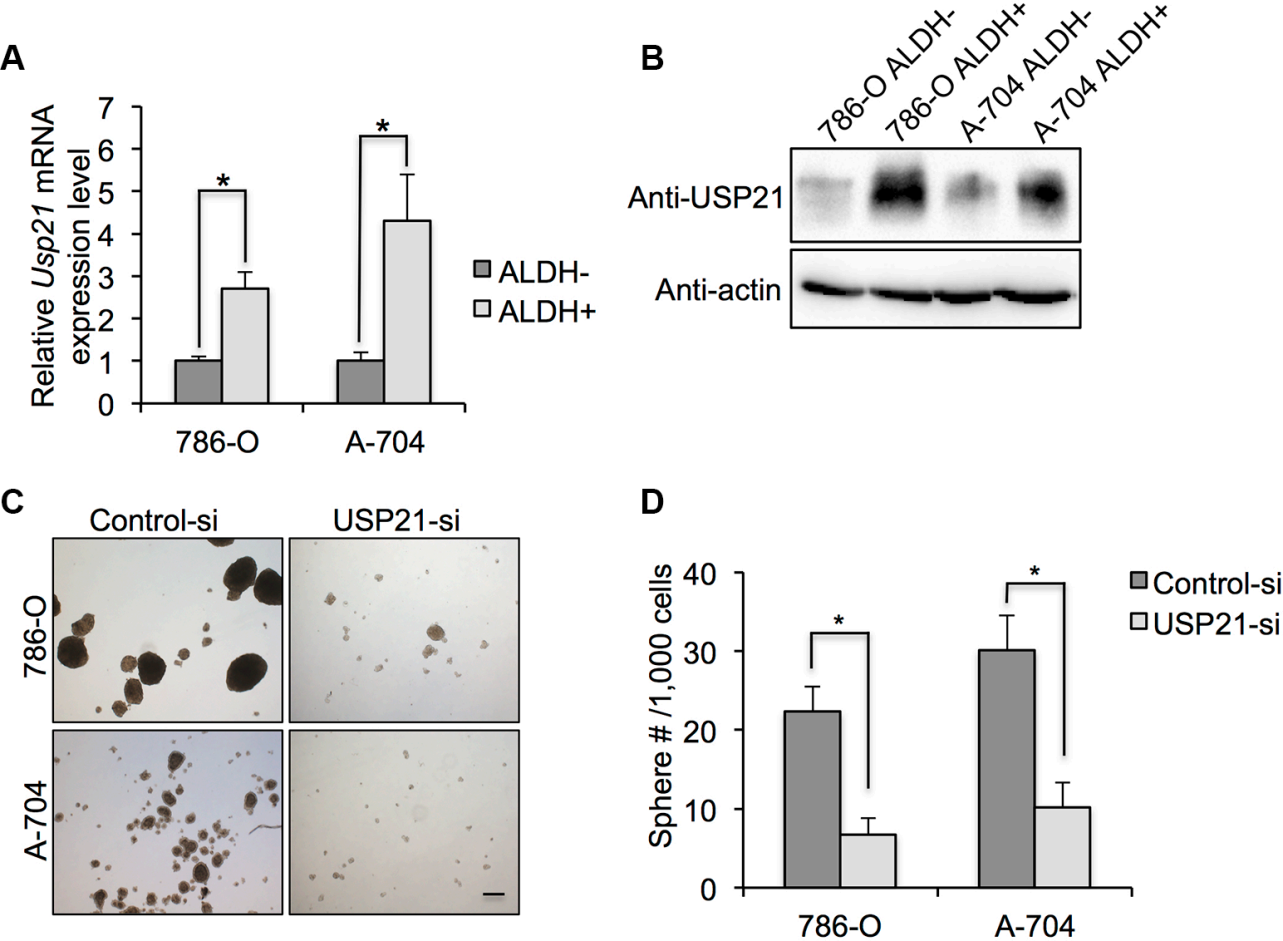
(**A**) qRT-PCR was used to detect the mRNA expression of IL-8 in ALDH^high^ and ALDH^low^ 786-O and A-704 cells. (**B**) Western blotting was used to detect the protein expression of IL-8 in ALDH^high^ and ALDH^low^ 786-O and A-704 cells. (**C**) Representative images of sphere formation assay in 786-O and A-704 cells. After sorting ALDHhigh cells, they were treated with control or USP21 siRNA and subjected to sphere formation assay. Scale bar, 100 μm. (**D**) Number of spheres formed by ALDHhigh cells treated with control or USP21 siRNA.

We asked if USP21 expression was enriched in the CSCs of RCC cell lines. As expected, our qRT-PCR and Western blot results showed that both mRNA and protein expression level of USP21 were higher in ALDH^high^ than in ALDH^low^ cell populations (Figure 4A and 4B), demonstrating that higher USP21 levels are associated with cells reported to possess CSC-like properties. Next, we examined whether USP21 maintain the self-renewal properties of CSCs. CSCs are capable of self-renew and forming spheres when grown under non-adherent serum free media. Therefore, to further establish the role of USP21 in CSCs growth and maintenance, we investigated the sphere formation capabilities of FACS-sorted CSCs treated with either control or USP21 siRNA. In the control cells, we observed 22.3 spheres/ 1,000 cells and 30.1 spheres/ 1,000 cells in the 786-O and A-704 cells, respectively. In contrast, only 6.7 spheres/1,000 cells and 10.2 spheres/1,000 cells in the corresponding USP21 knockdown lines (Figure 4C and 4D).

### Overexpression of USP21 increase the cell growth, invasion and cancer stem cell percentage of 786-O and A-704 cells

**Figure 5:**
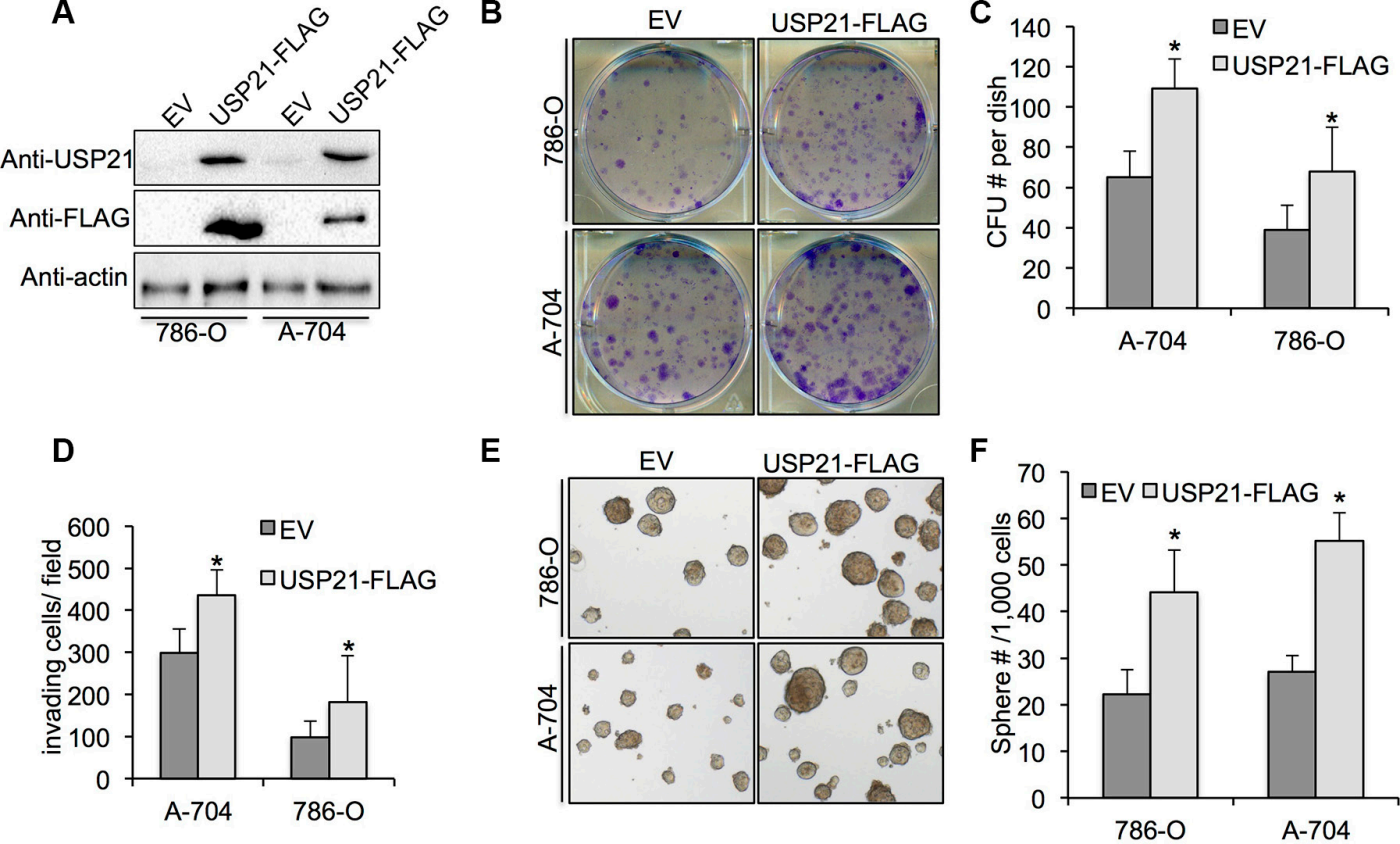
(**A**) 786-O and A-704 cells transfected with retrovirus carrying empty vector or USP21-FLAG-HA were tested for the presence of USP21 protein or FLAG-tag by Western blot. (**B, C**) Colony formation assay in 786-O and A-704 cells transfected with transfected with retrovirus carrying empty vector or USP21-FLAG-HA. Values are expressed as the mean ± SD. (**D**) Quantification of invasion assay of 786-O and A-704 cells transfected with transfected with retrovirus carrying empty vector or USP21-FLAG-HA. (**E, F**) Number of spheres formed in 786-O and A-704 cells transfected with transfected with retrovirus carrying empty vector or USP21-FLAG-HA.

To investigate whether overexpression of USP21 can promote the tumorigenic abilities of RCC cell lines, we introduced exogenous USP21 using retrovirus infection. Western blot using antibodies against USP21 and FLAG showed significant increased expression of USP21 proteins in both 786-O and A-704 cells transfected with USP21-FLAG-HA compared with empty vector (Figure 5A). We performed colony formation assays and showed that overexpression of USP21 markedly increase colony numbers (Figure 5B and 5C). Moreover, we observed an increase of invasion ability in RCC cell lines transfected with USP21-FLAG-HA compared with empty vector (Figure 5D). In addition, sphere formation capabilities of RCC cells transfected with USP21-FLAG-HA were drastically higher compared with controls (Figure 5E and 5F). Based on these results, we sought to further define the role of USP21 in regulating CSC property in RCC cell lines.

### USP21 is required for IL-8 expression in RCC

**Figure 6:**
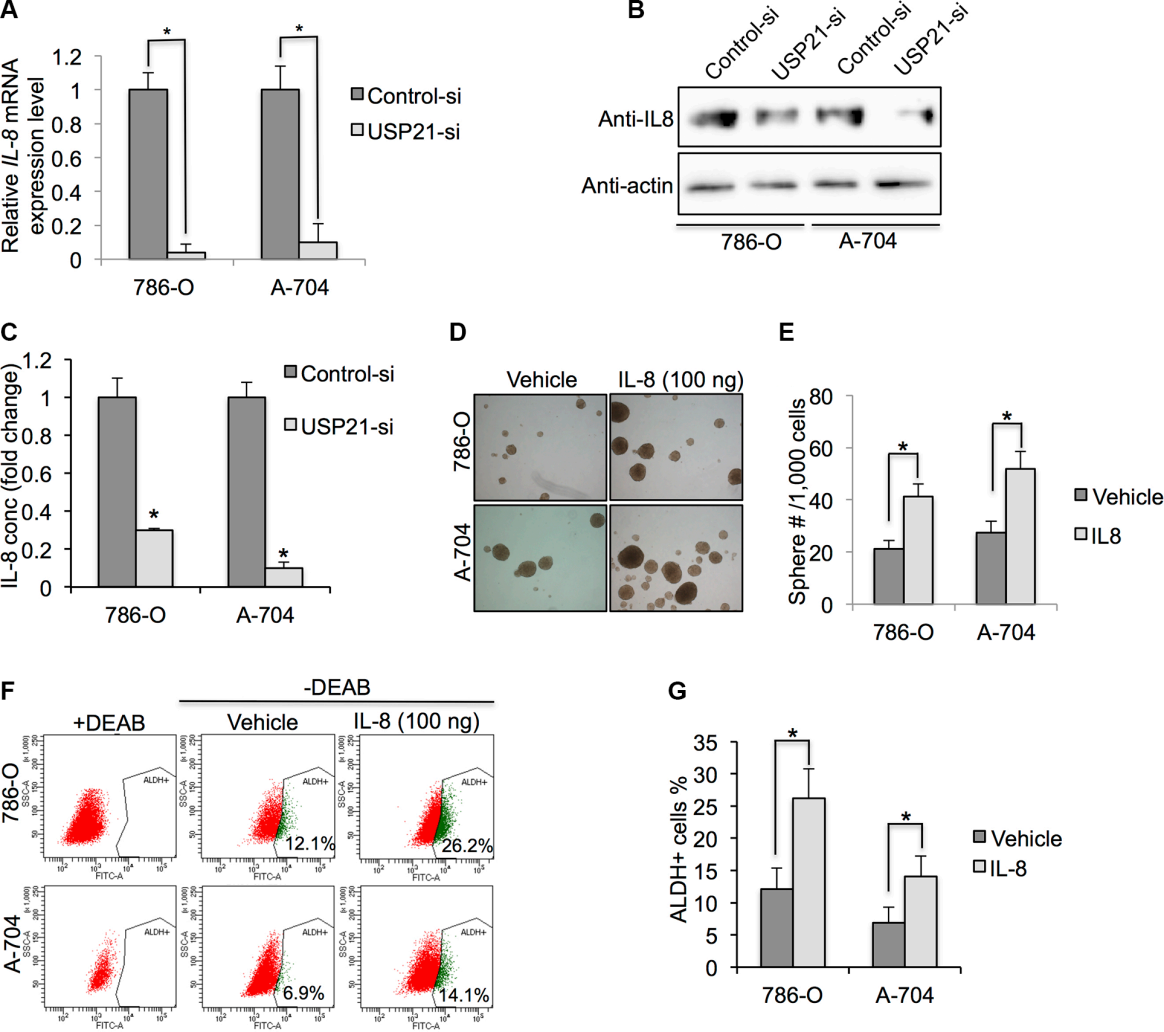
(**A**) qRT-PCR was used to detect the mRNA expression of IL-8 in 786-O and A-704 cells treated with control or USP21 siRNA. (**B**) Western blotting was used to detect the protein expression of IL-8 in 786-O and A-704 cells treated with control or USP21 siRNA. (**C**) ELISA assay of IL-8 in culture medium of 786-O and A-704 cells treated with control or USP21 siRNA. (**D**) Representative images of sphere formation assay in 786-O and A-704 cells treated with 100 ng/ml IL-8. (**E**) Number of spheres formed in 786-O and A-704 cells treated with 100 ng/ml IL-8. (**F, G**) ALDH^high^ cell percentage in 786-O and A-704 cells treated with control vehicle or 100 ng/ml IL-8.

Independent studies have shown a strong correlation between high Interleukin-8 (IL-8) levels and cancer stem cell-like properties in various human cancers [19–21]. However, whether IL-8 expression level is associated with USP21 and CSCs property in RCC cells remains undefined. Intriguingly, we found that there was significant difference in mRNA expressions of *IL-8* gene between control and USP21 siRNA RCC cells lines (Figure 6A). Consistent with mRNA level changes, using Western Blot analyses we also found knockdown of USP21 could evidently down-regulate IL-8 in RCC cells (Figure 6B). Since IL-8 is a secreted cytokine, we quantified the concentration of IL-8 in the culture medium using ELISA kit. Our results showed that knockdown of USP21 led to decrease of IL-8 secretion in RCC cell lines (Figure 6C). To establish whether IL-8 can regulate CSCs activity in RCC cell lines, we stimulated both 786-O and A-704 cells with IL-8 (100 ηg/ml) and measured the sphere formation ability and percentage of ALDH^high^ population (Figure 6D). We found 21.2 spheres/1,000 cells and 27.3 spheres/ 1,000 cells in the 786-O and A-704 cells, respectively. In contrast, we found 41.2 spheres/1,000 cells and 51.8 spheres/ 1,000 cells in the corresponding cells treated with IL-8 (Figure 6E). In addition, we observed 12.1% and 6.8% of ALD^Hhigh^ cells in the 786-O and A-704 cells, respectively. In contrast, 26.2% and 14.1% ALD^Hhigh^ cells were detected in the corresponding cells treated with IL-8 (Figure 6F and 6G). In summary, our results demonstrate that USP21 can regulate CSCs growth and self-renewal via the IL-8 signaling pathway.

### USP21 binds to IL-8 promoter

**Figure 7:**
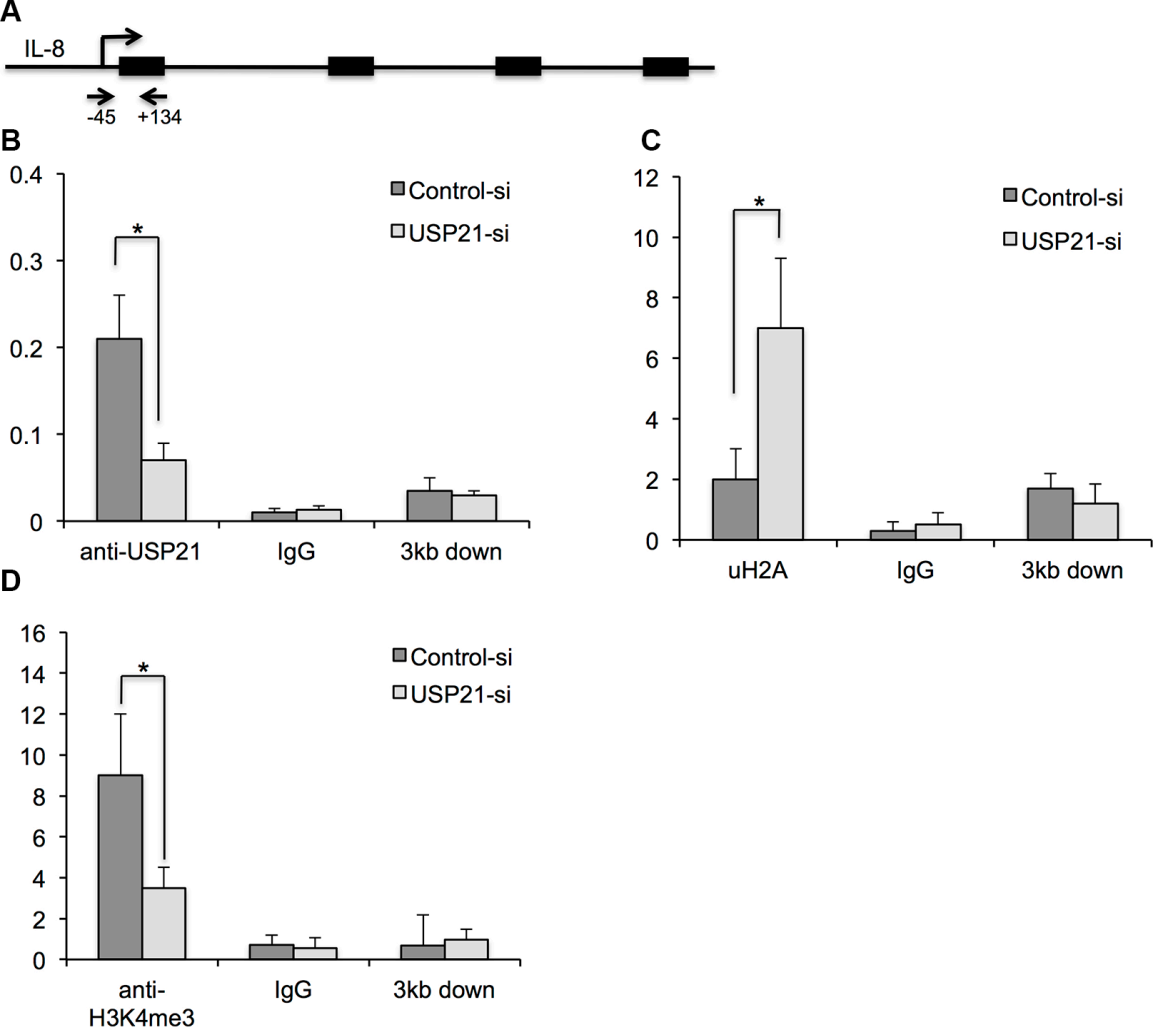
(**A**) Schematic showing the location of the amplicon (Arrows) used to detect ChIP-enriched fragments over the IL-8 promoter. (**B**–**D**) Analysis of binding of USP21, H2K199ub and H3K4Me3 modifications in the IL-8 promoter region or 3 kb downstream in 786-O cells depleted of USP21 compared with control by ChIP. 786-O cells were transfected with control or USP21 siRNA. IgG antibody was used as a ChIP control. Data are presented as the mean ± SD.

USP21-mediated histone H2A ubiquitylation can affects transcriptional initiation through its impact on H3K4 di- and trimethylation [14]. To determine whether USP21 regulates the transcriptional expression *IL-8* through binding to its promoter, we performed chromatin immunoprecipitation (ChIP) assays. Our results showed that USP21 was detected on the promoters of *IL-8* and USP21 knockdown significantly reduced the binding of USP21 to the promoter of *IL-8* in 786-O cells (Figure 7A). As expected, decreased binding of USP21 to the promoter regions of *IL-8* was associated with increased uH2A level and decrease of H3K4me3 levels (Figure 7B and 7C), confirming the function of USP21 in regulating the transcriptional activities of *IL-8*.

## DISCUSSION

Researches on CSCs in solid tumors have shown important findings over the last decade. In this study, we showed that USP21 is enriched in CSCs population of RCC. Knockdown of USP21 impaired self-renewal capacities of CSCs. USP21 can catalyze the hydrolysis of uH2A, which specifically represses the di- and trimethylation of H3K4 [14]. The IL family proteins are important regulators of inflammation [22]. In current study, we showed that USP21 binds to the promoter region of *IL-8* gene and regulates its transcriptional activity. Collectively, our data indicate that USP21 is an important regulator of CSC activity in RCC.

USP21 has been shown to interact with GATA3 to promote its stability via deubiquitination, which in turn increase the transcription FOXP3, a master transcriptional regulator of nTreg cells [23]. In addition, USP21 plays an important role in the down-regulation of TNFα-induced NF-κB activation through deubiquitinating RIP1 [19]. The protein stability of IL-33 is maintained by USP21 through deubiquitination [16]. In current study, we found that depletion of USP21 significantly repress the mRNA expression of IL-8 in RCC cell lines, confirming an essential role of USP21 in regulating inflammation pathway. Study has shown that IL-8, through its receptors CXCR1/2, is an important regulator of breast CSC activity [20]. Moreover, enhanced levels of IL-8 and its receptors CXCR1 and CXCR2 enhanced CSC migration, growth and stemness properties in glioblastoma [24]. Recently, IL-8 was shown to be involved in the renewal of colorectal cancer stem cells and an IL-8 neutralizing antibody was shown to inhibit sphere formation *in vitro* as well as colon cancer growth *in vivo* [25]. Our study demonstrates that IL-8 affects the CSCs activity in RCC and how IL-8 activity is mediated by USP21 through its binding to the promoter region. However, whether USP21 can regulate the protein stability of IL-8 needs further investigation.

In conclusion, the present study provides evidence demonstrating that USP21 is significantly upregulated in CSCs of RCC cell lines. In addition, it was demonstrated that USP21 directly targets IL-8 in RCC cells. The results of the present study support USP21/IL-8 pairs as novel diagnostic or therapeutic target for RCC.

## MATERIALS AND METHODS

### Cell lines and siRNA transfection and virus transduction

The HEK293T and human RCC cell lines 786-O, 769-P, A-704, Caki-1 and Caki-2 were maintained in DMEM (Invitrogen) supplemented with 10% FBS (Gibco) and 1% Penicillin Streptomycin (Sigma-Aldrich). RCC cells were transfected with siRNAs using Lipofectamine RNAiMax (Invitrogen) following manufacturer's guidance. Two siRNA sequences are listed: 5′-GCUAGAAGAACCUGAGUUA-3′; 5′-GAGCUGUCUUCCAGAAAUA-3′. Retrovirus was generated by co-transfection of Flag-HA-USP21 (addgene) or empty vector with packaging plasmids into HEK293T cells using Lipofectamine 2000 following manufacturer's instruction. To obtain stable cell lines, RCC cells transfection with retrovirus were selected with antibiotics for 7 days.

### Cell proliferation assay, colony forming assay and invasion assay

Cell proliferation assay, colony-forming assay and invasion assay were performed as previously described [26]. After transfected with control or USP21 siRNA, 2.5 × 10^3^ 786-O or A-704 cells were seeded in 96-well plates. Cell proliferation was measured every day for 6 days by an MTT [3-(4,5-dimethylthiazol-2-yl)-2,5-diphenyltetrazolium bromide] assay (Thermo Fisher Scientific) using the manufacturer's guidance. For colony forming, single cells were seeded onto 6-well plates (Corning) at a density of 1000 cells/well and then incubated at 37°C. After two weeks of culture, colonies were stained with 0.5% crystal violet solution for 30 min at room temperature. The colonies from three replicate wells were counted, and pictures were captured digitally. To examine the cell invasion, 1 × 10^5^ siRNA-transfected cells were plated on Matrigel invasion chambers (Corning, NY, USA). Invaded cells were stained with the HEMA-3 kit (Fisher) after 24 hours. Cell numbers counted and averaged under microscope.

### Western blot analysis and IL-8 ELISA

Western blot analysis was performed as previously described [27]. The cell lysate with SDS sample buffer was separated by denaturing SDS-PAGE. Separated proteins were transferred onto nitrocellulose membranes. Membranes were blocked with 5% milk for 1 hour and incubated with primary antibodies overnight, including anti-USP21 antibody (Santa Cruz, 1:1000), anti-IL-8 antibody (Abcam 1:1000). β-Actin was used a loading control and was detected with a mouse mAb (Sigma-Aldrich, 1:2000). The immunocomplexes were treated with horseradish peroxidase (HRP)-conjugated secondary antibodies (Beyotime Biotech) and detected using BeyoECL PLUS Kit (Beyotime Biotech). Cultured carcinoma cells A-704 and 786-O treated with Control or USP21 siRNA were plated on a 100 mm plate. IL-8 concentration was measured using the IL-8 ELISA kit (R&D systems). All assays were preformed as to the manufactures specifications.

### RNA extraction and quantitative real time reverse transcriptase polymerase chain reaction

Total RNA was isolated from cells using TRIZOL reagent (Invitrogen), according to manufacturer's instructions. RNA was quantified spectrophotometrically using Nanodrop. Real-time RT-PCR was performed using SsoFast Evagreen Super mix (Bio-Rad). Cycling conditions followed the manufacturer's instructions, and CFX Manager was used for analyses (Bio-Rad). Expression levels were normalized to glyceraldehyde-3-phosphate dehydrogenase (GAPDH) expression, as previously described [28]. The primers used were as follows:

GAPDH: forward 5′-TGTTAGCTGATGCCGAC TTG-3′;

reverse 5′-TTCTTAGCCCGCTCAACACT-3′;

USP21: forward 5′-CAGGTCTGCCTGATGA ACGG-3′;

reverse 5′-GCTAAGTTGGTCCGAGATGGG-3′;

IL-8: forward 5′-TTTTGCCAAGGAGTGCTAA AGA-3′;

reverse 5′-AACCCTCTGCACCCAGTTTTC-3′.

### Aldefluor assay, fluorescence-activated cell sorting (FACS) and sphere formation assay

Cells treated with control or USP21 siRNA were assayed for aldehyde dehydrogenase (ALDH) activity using the Aldefluor kit (Stem Cell Technologies) according to the manufacturer's instructions. FACS was performed on a BD FACSAria III (BD Biosciences) cell-sorting system. Data was analyzed with the FlowJo software (Tree Star, Inc). Isolated ALDH^high^ cells from the 786-O and A-704 cell lines (1,000 cells/dish) were cultured in serum-free medium including 10 ng/mL epidermal growth factor (EGF) (Thermo Fisher Scientific) and 20 ng/mL basic fibroblast growth factor (bFGF) (Thermo Fisher Scientific) in ultra-low-attachment 6-well plates (Corning Inc., Corning, NY, USA) for 1 week. Formation of sphere was assessed by counting the number of spheres (> 50 mm) under microscope. Five replicates were used for each condition, and the experiment was repeated two times.

### ChIP assays

ChIP assays were carried out using a ChIP assay kit following the manufacturer's protocol (Upstate Biotechnology). Cells were treated with a dimethyl 3,3′dithiobispropionimidate-HCl (DTBP) solution (Pierce) for 10 min at room temperature, followed by formaldehyde treatment for 15 min in a 37°C water-bath. For each ChIP reaction, 2 × 10^6^ cells were used. All resulting precipitated DNA samples were quantified with a specific set of primers for individual genes using real-time PCR. For the IL-8 promoter, primers for amplification of the USP21 binding, uH2A and H3K4me3 status (nucleotides −134 to +45) were CATCAGTTGCAAATCGTGGA and GTTCCTTCCGGTGGTTTCTT. For the non-specific IL-8 promoter fragment (nucleotides +2976 to +3132), primers were CAGCCAAAACTCCACAGTCA and TTTGGAGAGCACATAAAAACATC. ChIP-qPCR results were calculated as % input. All experiments were performed at least three times and each experiment in triplicate.

### Statistical analysis

Student's *t*-test was used to comparing the means of two samples. A value of *p* less than 0.05 (*P < 0.05) was regarded statistically significant.

## SUPPLEMENTARY MATERIALS


