# Supplementary material for: Ubiquitin specific peptidase 21 regulates interleukin-8 expression, stem-cell like property of human renal cell carcinoma
Source: Oncotarget. 2016 May 31;7(27):42007–16. doi: 10.18632/oncotarget.9751 (PMC5173112; doi:10.18632/oncotarget.9751)
Supplement: Supplementary file 1 [file oncotarget-07-42007-s001.pdf]

# Ubiquitin specific peptidase 21 regulates interleukin-8 expression, stem-cell like property of human renal cell carcinoma

## Supplementary Materials

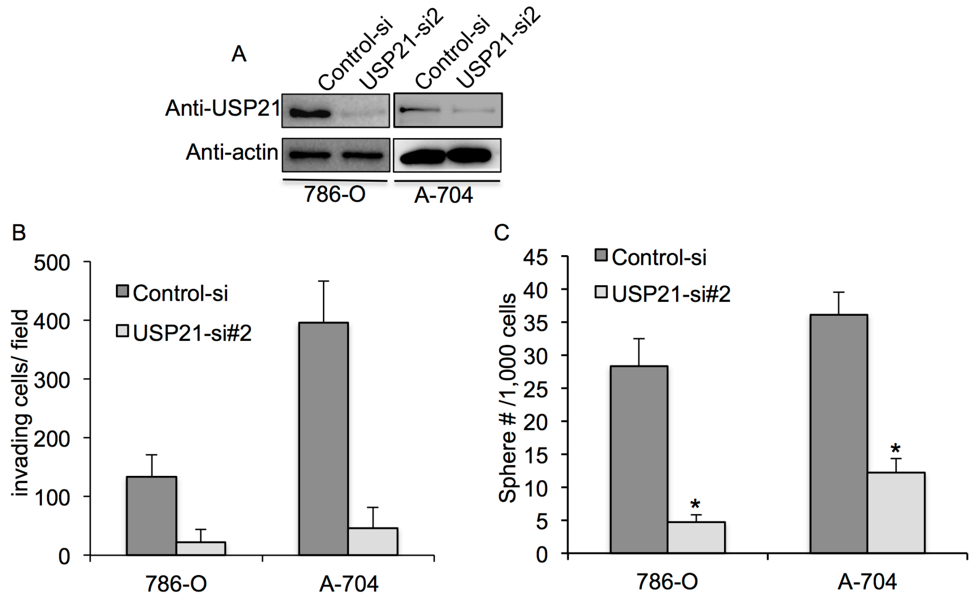


**Supplementary Figure S1:** (**A**) 786-O and A-704 cells transfected with control siRNA or USP21 siRNA#2 for 48 hours were tested for the presence of USP21 protein by Western blot. (**B**) Invasion assay of 786-O and A-704 cells transfected with control siRNA or USP21 siRNA#2. (**C**) Number of spheres formed in 786-O and A-704 cells transfected with control siRNA or USP21 siRNA#2.
